# Supplementary material for: Causal Relationship between Aging and Anorexia Nervosa: A White-Matter-Microstructure-Mediated Mendelian Randomization Analysis
Source: Biomedicines. 2024 Aug 16;12(8):1874. doi: 10.3390/biomedicines12081874 (PMC11351342; doi:10.3390/biomedicines12081874)
Supplement: Supplementary file 1 [file biomedicines-12-01874-s001.zip › Supplementary Figure/Figure S4/Singe SNP plot of MR analysis for TL to ubm-b-1546.pdf]

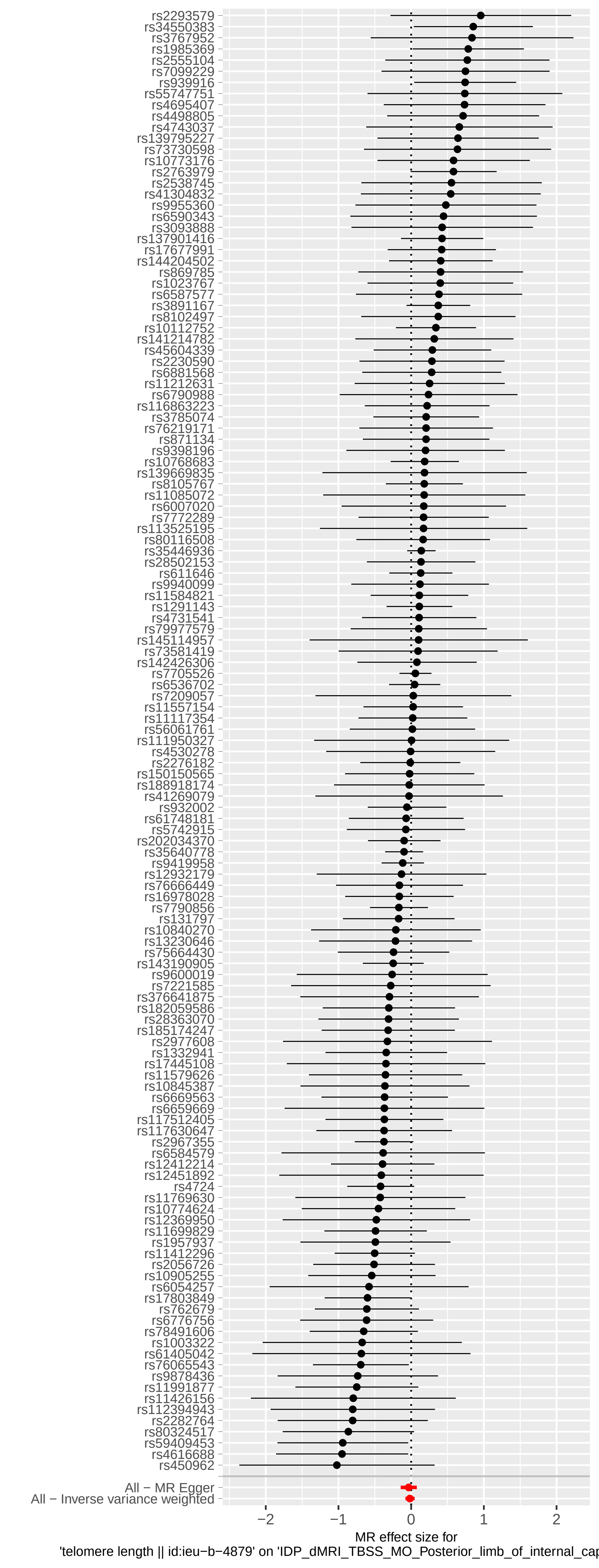
rs2293579
rs34550383
rs3767952
rs1985369
rs2555104
rs7099229
rs939916
rs55747751
rs4695407
rs4498805
rs4743037
rs139795227
rs73730598
rs10773176
rs2763979
rs2538745
rs41304832
rs9955360
rs6590343
rs3093888
rs137901416
rs17677991
rs144204502
rs869785
rs1023767
rs6587577
rs3891167
rs8102497
rs10112752
rs141214782
rs45604339
rs2230590
rs6881568
rs11212631
rs6790988
rs116863223
rs3785074
rs76219171
rs871134
rs9398196
rs10768683
rs139669835
rs8105767
rs11085072
rs6007020
rs7772289
rs113525195
rs80116508
rs35446936
rs28502153
rs611646
rs9940099
rs11584821
rs1291143
rs4731541
rs79977579
rs145114957
rs73581419
rs142426306
rs7705526
rs6536702
rs7209057
rs11557154
rs11117354
rs56061761
rs111950327
rs4530278
rs2276182
rs150150565
rs188918174
rs41269079
rs932002
rs61748181
rs5742915
rs202034370
rs35640778
rs9419958
rs12932179
rs76666449
rs16978028
rs7790856
rs131797
rs10840270
rs13230646
rs75664430
rs143190905
rs9600019
rs7221585
rs376641875
rs182059586
rs28363070
rs185174247
rs2977608
rs1332941
rs17445108
rs11579626
rs10845387
rs6669563
rs6659669
rs117512405
rs117630647
rs2967355
rs6584579
rs12412214
rs12451892
rs4724
rs11769630
rs10774624
rs12369950
rs11699829
rs1957937
rs11412296
rs2056726
rs10905255
rs6054257
rs17803849
rs762679
rs6776756
rs78491606
rs1003322
rs61405042
rs76065543
rs9878436
rs11991877
rs11426156
rs112394943
rs2282764
rs80324517
rs59409453
rs4616688
rs450962
All − MR Egger
All − Inverse variance weighted
−2
−1
0
1
2
MR effect size for
'telomere length || id:ieu−b−4879' on 'IDP_dMRI_TBSS_MO_Posterior_limb_of_internal_cap
